# Evaluation of Biochemical Parameters in *Caretta caretta* Sea Turtles

**DOI:** 10.3390/vetsci11110571

**Published:** 2024-11-16

**Authors:** Rosaria Disclafani, Paola Galluzzo, Giorgia Schirò, Irene Vazzana, Chiara Lomonaco, Vincenzo Monteverde, Salvatore Dara

**Affiliations:** 1 Centro di Referenza Nazionale sul Benessere, Monitoraggio, Diagnostica delle Malattie delle Tartarughe Marine (C.Re.Ta.M.), Istituto Zooprofilattico Sperimentale della Sicilia “A. Mirri”, 90129 Palermo, Italy; rosaria.disclafani@zssicilia.it (R.D.); irene.vazzana@izssicilia.it (I.V.); chiara.lomonaco1@studenti.unime.it (C.L.); vincenzo.monteverde@izssicilia.it (V.M.); salvatore.dara@izssicilia.it (S.D.); 2 Centro di Sostenibilità e Transizione Ecologica, University of Palermo, 90133 Palermo, Italy; 3 Department of Veterinary Sciences, University of Messina, 98169 Messina, Italy

**Keywords:** *Caretta caretta*, biochemical parameters, Mediterranean Sea, sea turtles, loggerhead sea turtle

## Abstract

The *Caretta caretta* is the only known sea turtle that nests along the coasts of the Mediterranean Sea. It is considered a vulnerable species by the International Union for Conservation of Nature (IUCN) because it is threatened by human activities. The aim of this work was to analyze biochemical parameters in different age groups of *C. caretta* subjects recovered at the Centro di Referenza Nazionale sul Benessere, Monitoraggio e Diagnostica delle Malattie delle Tartarughe Marine (C.Re.Ta.M.), Sicily. Biometric parameters were recorded for each subject. Peripheral blood was collected and centrifuged, and 18 biochemical parameters were analyzed. Glucose and total proteins showed lower values in juvenile turtles than in sub-adult and adult subjects. Since blood biochemical parameters can be influenced by several factors (age, sex, infectious disease, or trauma), we evaluated differences in the values of some parameters between the reproductive and non-reproductive seasons of adult turtles whose sex and cause of stranding could be determined. Despite the small number of subjects, it would seem that, in adult turtles, the values of ALP, γ-GT, Fe, and LDH are higher during the reproductive season. These biochemical variations showed important differences in the parameters, underlining their importance in assessing the health status of turtles and better understanding their physiology during different stages of their lives.

## Simple Summary

Sea turtles are vital components of marine ecosystems, and their conservation has become a global concern due to declining populations. Monitoring the health of sea turtles is essential for their management, and blood biochemical parameters are increasingly being used as a tool to assess their physiological state. These parameters provide crucial information on metabolic, nutritional, and immune statuses, which are integral to understanding the health, reproductive condition, and stress levels of these animals. The aim of this study was to assess blood biochemical parameters at various ages and during the reproductive and non-reproductive period of *Caretta caretta* sea turtles, the only known species nesting on the Mediterranean coast. Eighteen biochemical blood parameters were analyzed on sixty-seven subjects. The glucose and total protein values showed significant differences between juveniles, sub-adults, and adults. Despite the small number of subjects, it would seem that, in adult turtles, the values of ALP, γ-GT, Fe, and LDH are higher during the reproductive season. These results made it possible to take a snapshot of the values of the most common biochemical parameters, highlighting the importance of monitoring these to assess the health status and physiological period of *Caretta caretta* turtles.

## 1. Introduction

The *Caretta caretta* sea turtle is a long-lived marine species widely distributed along the temperate and tropical zones of all oceans, as well as in the entire Mediterranean Sea [1,2], and the only known species nesting along the Italian coast [3]. This species is classified in the IUCN Red Listas a vulnerable species [4], because it is highly threatened by human activities: incidental capture by fishing gears [5], entanglement, impact with boats, and foreign body ingestion, including plastic, hooks, and fish lines, compromising their lives [6]. Since the *C. caretta* is considered a bio-accumulative, as well as an excellent bioindicator [7,8] and sentinel species [9], it is crucial to evaluate its health status to monitor and define the status of the marine environment. Therefore, the evaluation of blood and biochemical parameters is fundamental because it reflects both animals’ physiological conditions and environmental characteristics [10]. The body condition of various reptiles, including sea turtles, may influence their stress response based on their metabolic pattern.

Although many studies have reported ranges for healthy sea turtles, the ranges of biochemical values for healthy turtles may be due to their geographic location, diet, size and sex, breeding season, and health status [11]. Biochemical parameters are a useful diagnostic tool in animal health management [2] in order to diagnose possible diseases as well as verifying and evaluating the health status of each subject [12]. Blood profiles have already been successfully used to diagnose chelonian diseases and can also be used to assess the physiological status of populations.

Sea turtles are exposed to a variety of environmental stressors, such as habitat degradation, pollution, climate change, and bycatch in fisheries. These stressors can lead to physiological imbalances that may be reflected in an altered blood biochemistry. For instance, changes in blood glucose, protein, electrolyte, and enzyme levels can provide valuable insights into the stress response of sea turtles to environmental and anthropogenic threats [12].

Moreover, variation in some biochemical parameter values during the reproductive season was reported [5].

Consequently, it has been considered of high priority to establish the species-specific ranges of biochemical profiles of the *C. caretta* species [13], also due to the scarce data in the literature on this marine species.

The aim of this work is to evaluate the reference ranges of biochemical parameters of *C. caretta* subjects recovered from 2022 to 2024 at the Centro di Referenza Nazionale sul Benessere, Monitoraggio e Diagnostica delle Malattie delle Tartarughe Marine (C.Re.Ta.M.) located in Palermo, Sicily, Italy. Moreover, we focused on investigating the presence of eventual differences in biochemical parameters between different age stages.

## 2. Materials and Methods

### 2.1. Subjects

Sixty-seven *C. caretta* subjects were recovered at C.Re.Ta.M. at the Istituto Zooprofilattico Sperimentale della Sicilia “A. Mirri” (Italy) between 2022 and 2024. All the sea turtles arrived at the center for different, often concomitant reasons, such as traumatic injuries (n.11), the presence of hooks and lines in their gastrointestinal tract (n.41), and plastic ingestion (n.21).

### 2.2. Morphological Parameter Collection

Carapace length (CCL), width (CCW), and total body weight (TBW) were measured. Sex, when possible, was identified by visual inspection. Additionally, the body condition index (BCI) (BCI = (mass * 10,000)/SCL3) and the straight carapace length (SCL = −1.442 + (0.948 × CCL)) were calculated [14,15]. Sea turtles of the *C. caretta* species were classified into three different age classes: juveniles (21–40 cm CCL), sub-adults (41–65 cm CCL), and adults (>65 cm CCL) [16].

### 2.3. Blood Biochemical Analysis

Blood samples from the sea turtles were collected upon arrival at the C.Re.Ta.M., and the values of the first sampling were considered for this study. Peripheral venous blood was sampled and centrifuged at 3500 rpm (1500 rcf) for 10 min for serum collection.

Sera were then analyzed by the multiparametric chemistry analyzer BS-480 Mindray, an optical system (340 nm–800 nm). The biochemical panel included glucose (Glu), urea, total bilirubin (Tot-Bil), total protein (Tot. P), albumin (Alb), alkaline phosphatase (ALP), alanine aminotransferase (ALT), aspartate aminotransferase (AST), creatinine kinase (CK), lactate dehydrogenase (LDH), calcium (Ca), phosphorus (P), sodium (Na), potassium (K), chlorine (Cl), iron (Fe), magnesium (Mg), and gamma GT (GGT). A calibrator (Multi Sera Calibrator, Mindray) and controls (ClinChem Multi Control (Level 1) and ClinChem Multi Control (level 2), Mindray) were used in each work session.

### 2.4. Statistical Analysis

Descriptive statistics (mean, standard deviation, median, minimum, and maximum) were performed with the use of Microsoft Excel (Microsoft Corporation, Redmond, WA 98052, USA). The comparison between the different age groups was carried out using a one-way ANOVA test. Data were statistically analyzed by the GraphPad software package (version 8.1), and statistical significance was accepted at *p*-value < 0.05.

## 3. Results

### 3.1. Morphological Parameters

Of the 67 *C. caretta* individuals stranded on the Sicilian coast, 31 were identified as juveniles, 25 as sub-adults, and 11 as adults (8 females and 3 males). The three males were hospitalized one per year (Table 1).

The biometric data of the three males were as follows: CCL 52, 56, and 71 cm; TBW 16.3, 21, and 41.6 Kg; BCI 1.49, 1.54, and 1.46; and SCL 47.85 cm, 51.6 cm, and 65.87 cm.

Because of the small number of males, these data were analyzed individually and were not considered in the statistical analysis.

### 3.2. Blood Biochemical Parameter Analysis

A total of 18 biochemical blood parameters were measured: sodium (Na), potassium (K), chlorine (Cl), glucose (Glu), alanine aminotransferase (ALT), aspartate aminotransferase (AST), phosphorus (P), magnesium (Mg), calcium (Ca), alkaline phosphatase (ALP), gamma GT (GGT), total protein (Tot. P), albumin (Alb), urea, creatinine kinase (CK), iron (Fe), lactate dehydrogenase (LDH), and total bilirubin (Tot-Bil).

The mean values obtained for all groups are reported in Table 2 (juveniles), Table 3 (sub-adults), and Table 4 (adults).

The biochemical data of the male subjects are reported in Table S1.

**Figure 1:**
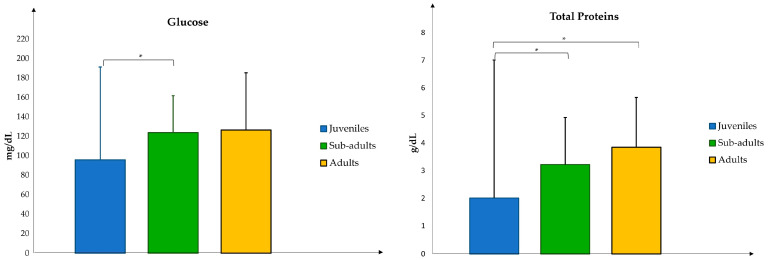
Comparison of biochemical data in the three age groups of *C. caretta* turtles. The asterisk indicates a *p*-value < 0.05.

Among all the analytes evaluated, Glu and TP showed significant differences between the age groups considered (Figure 1). In particular, Glu was less abundant in juvenile subjects than in sub-adults (*p*-value: 0.02), while TPs were less abundant in juvenile subjects than in sub-adult (*p*-value: 0.01) and adult (*p*-value: 0.01) turtles.

### 3.3. Blood Biochemical Parameters and Reproductive Season

The observation of biochemical parameter data of adult females of *Caretta caretta* recovered at the C.Re.Ta.M. was also made based on the two periods during which they had become stranded: reproductive (from April to September) or non-reproductive season (from October to March).

Most of the turtles hosted by the center were recovered during the reproductive season. In particular, 26/31 juveniles, 24/25 sub-adults, and 4/8 adult subjects.

Since the biochemical parameter values analyzed in this study can be influenced by different factors (sex, age, health status), only the data from adult turtles were evaluated. Indeed, despite the small number of subjects, we knew that they were all females and had ingested hooks and lines and/or plastic.

Data on the blood biochemical parameters of adult turtles are reported in Table 5.

**Figure 2:**
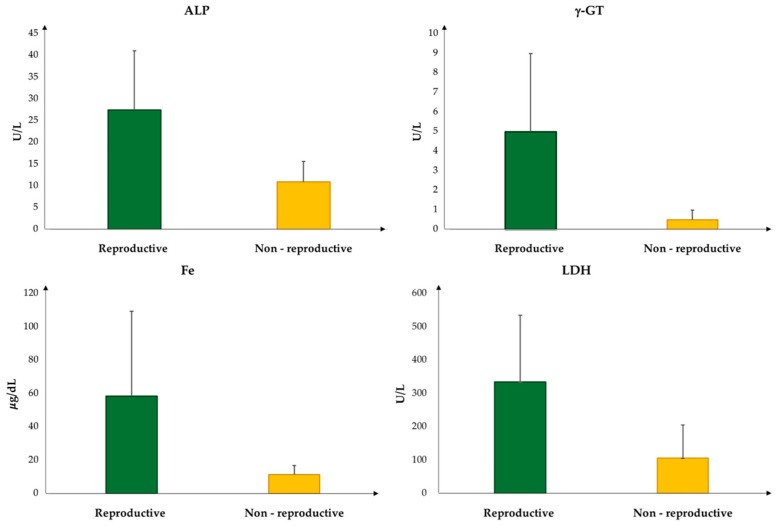
Main differences in the biochemical values of *C. caretta* adult turtles between the reproductive and non-reproductive season.

Given the small number of subjects, no statistical analysis was performed. However, based on data observation, it is likely that ALP, γ-GT, Fe, and LDH values are generally higher during the reproductive season (Figure 2).

## 4. Discussion

In this study, we focused on evaluating the blood biochemical parameters of *C. caretta* turtles that were recovered at the C.Re.Ta.M. in different health conditions. As it is a rescue center, no healthy sea turtles are usually recovered. Indeed, a limitation of this study is that all rescued sea turtles recovered at the center showed pathologic conditions of various nature. However, this study contributes to the definition of value ranges of blood biochemical parameters that could be likely observed in stranded sea turtles of different ages. Nevertheless, blood biochemical parameters are essential to monitor the recovery of sea turtles undergoing rehabilitation.

Blood biochemical parameters can vary significantly across different life stages. For instance, juvenile and adult sea turtles may exhibit different biochemical profiles due to variations in growth rates, diet, and metabolism [17].

In this study, the values of all blood biochemical parameters analyzed showed no significant differences between the three age groups studied, except for glucose and total proteins.

Blood glucose is a key indicator of energy metabolism and can reflect the nutritional and environmental conditions and stress status of sea turtles [17]. A significant increase in glucose in sea turtle blood after capture or transport was reported [18]. Furthermore, variations in glucose levels can be influenced by environmental temperature, with higher temperatures often associated with an elevated blood glucose due to increased metabolic rates [12]. Meanwhile, due to their smaller body size, juvenile sea turtles have an increased susceptibility to a severe or rapid decrease in water temperature. Lower glucose values in juvenile turtles than in sub-adults were observed in this study, which could have been influenced by the aforementioned factors: the season in which the turtles had been recovered and the duration of the starving period.

The total protein values considered in this study increased progressively in the three groups: the lowest values were recorded in juveniles (2.01 ± 1.44 g/dL), intermediate values in sub-adults (3.22 ± 1.73 g/dL), and higher values in adults (3.85 ± 1.88 g/dL). This increase is in agreement with another study [19] in which an increase in Tot. P values was associated with size, dietary changes, physiological changes associated with vitellogenesis, and somatic increases.

Seasonal changes in water temperature, food availability, and reproductive cycles can influence the blood biochemistry of sea turtles. During the warmer months, metabolic rates increase, leading to higher levels of glucose, protein, and enzymes in the blood. Conversely, during colder months or periods of fasting, turtles may exhibit reduced metabolic activity and lower biochemical values. For females, reproductive activity, particularly the production of eggs, can lead to elevated calcium and phosphorus levels, reflecting the increased demand for these minerals during shell formation [20].

In this study, between the reproductive and non-reproductive season, differences in calcium and phosphorus levels were not observed, but other values seemed to vary.

Since the biochemical parameter values analyzed in this study can be influenced by different factors (infectious disease or injury) and we had a small number of adult subjects, our observations could add valuable new data to the literature about this marine species. A statistical analysis was performed. The data show that the values of ALP, γ-GT, Fe, and LDH were higher during the reproductive season.

High ALP levels have often been associated with increased plasma activity of AST and ALT and could suggest malnutrition or a prolonged diet of non-natural foods, causing liver disorders in captive turtles [21]. Although in this study the AST and ALT values were not significantly different between the two seasons, the ALP values were higher during the reproductive season (27.25 ± 13.6 U/L) compared to the non-reproductive season (11 ± 4.7 U/L). An increase in ALP values might suggest malnutrition or a prolonged diet of non-natural foods which can cause liver disorders in captive turtles [21]. Indeed, all subjects hospitalized at the center had ingested plastic, hooks, and lines, supporting this hypothesis.

The literature is poor in terms of relating γ-GT activity to the reproductive cycle in reptiles because some authors claim that γ-GT values have barely detectable concentrations in reptile serum [22]. However, an increase in γ-GT values was observed in adult female sea turtles during the pre-nesting period [23] according to our study. Other authors [24] reported that the value of this enzyme increased during egg-laying activity compared to other reproduction periods.

The LDH enzyme is valuable in diagnosing conditions such as capture myopathy or trauma-related injuries: elevated LDH values were observed in stranded loggerhead turtles, suggesting muscle damage or stress [25]. In addition, the LDH levels showed a significant difference during the reproductive season, indicating potential liver, skeletal muscle, or heart muscle issues [25], potentially associated with anthropogenic factors (hooks, lines, and plastics).

Higher iron values during the reproductive season were reported; adult turtles had higher iron concentrations than juvenile turtles, and females had significantly higher iron concentrations than males [26]. Considering that high concentrations of iron are present in the yolk of loggerhead and green sea turtle eggs [27,28], it is possible that when females prepare their follicles, iron may be transferred to the yolk at high concentrations to support normal embryonic development. Iron concentrations during the reproductive period could be linked to folliculogenesis, as observed in alligators (*Alligator mississipiensis*), so that they may pass on adequate amounts to their offspring [29].

This study provides important information on the biochemical blood profile of *C. caretta* turtles in different age groups, showing how biochemical parameters differ between stranded juveniles, sub-adults, and adults. The present study aims to describe the existence of age-specific variations in glucose and total protein levels. These variations underline the importance of considering age in the health assessment and treatment of sea turtles. The impact of human activity, as suggested by the high levels of ALP in adults associated with the presence of anthropogenic litter, is a critical area of concern that requires immediate attention. These results highlight the importance of promoting sustainable practices and reducing marine pollution to protect *C. caretta* populations. This study provides a picture of the biochemical blood parameters of this sea turtle species when specimens are not healthy. Through the analysis of various biochemical indicators, we were able to delineate the physiological characteristics of these animals under suboptimal conditions, offering valuable insights for their monitoring and rehabilitation. Despite the challenges posed by the inherent variability among individuals and age groups, the results underline the importance of a multidisciplinary approach in sea turtle care and conservation. The discrepancies observed between different age groups and comparisons with existing data in the literature support the need for further research to refine current knowledge of the health status of *C. caretta* and improve intervention strategies.

To the best of our knowledge, this is the first study to relate changes in the biochemical parameters of *C. caretta* to the reproductive and non-reproductive seasons.

## 5. Conclusions

In conclusion, this study shows significant differences in Glu and TP between the age groups considered. Moreover, the ALP, γ-GT, Fe, and LDH values were higher during the reproductive season.

This study enriches the body of knowledge on sea turtle welfare, laying a solid foundation for future investigations and the development of more targeted and effective rehabilitation practices.

## Figures and Tables

**Table 1:** Morphometric data and body condition index of juvenile, sub-adult, and adult specimens of *C. caretta*.

	Juveniles (n.31)			Sub-Adults (n.25)			Adults (n.8)		
	Mean ± SD	Min	Max	Mean ± SD	Min	Max	Mean ± SD	Min	Max
**CCL (cm)**	31.04 ± 4.87	20.50	39.50	52.30 ± 6.98	41.50	63	68.62 ± 2.86	65	72
**SCL (cm)**	27.99 ± 4.62	17.99	36	48.14 ± 6.62	37.90	58.28	63.61 ± 2.71	60.18	66.81
**Mass (Kg)**	3.99 ± 1.51	1.10	7.50	19.79 ± 9.06	7.90	36.60	40 ± 6.88	31	53
**BCI**	1.78 ± 0.400	1.35	3.26	1.77 ± 1.05	1.14	6.72	1.54 ± 0.18	1.39	1.94

**Table 2:** Blood biochemistry data of the 31 *C. caretta* juvenile subjects.

Juveniles (n.31)						
Parameter	Mean (SD)	Median	Min	Max	10° Percentile	90° Percentile
**Na, mmol/L**	145.28 ± 32.38	152.70	2.90	171.10	142	164
**K, mmol/L**	3.43 ± 0.71	3.33	1.89	4.78	2.85	4.40
**Cl, mmol/L**	112.04 ± 15.59	115.20	60	131.50	104.50	126.17
**Glu, mg/dL**	95.67 ± 31.08	91.50	40	149	54.80	138.53
**ALT, U/L**	4.09 ± 5.65	2	0	26	0	10
**AST, U/L**	216.16 ± 151	156	1	664	81	410
**P, mg/dL**	7.07 ± 3.15	6.62	0.08	14.40	3.80	11
**Mg, mg/dL**	8.89 ± 11.21	4.20	1.10	38.25	1.96	26.16
**Ca, mg/dL**	6.22 ± 3.39	5.70	1.80	19.30	3.60	10.30
**ALP, U/L**	27 ± 28.79	21	1	153	3	49
**γ-GT, U/L**	1.03 ± 2.86	0	0	16	0	2
**TP, g/dL**	2.01 ± 1.44	1.60	0.40	6	0.60	4.60
**ALB, g/dL**	1.02 ± 1.17	0.70	0.10	6	0.20	1.70
**Urea, mg/dL**	165.20 ± 74	156	17.36	340.45	84.89	264.10
**CK, U/L**	1951.70 ± 2426.70	843	27	7914	69	7238
**Fe, ** **µg/dL**	30.90 ± 57.23	19	4	323	4	54
**LDH, U/L**	188.03 ± 222.20	129	8	1140	17	341
**Tot-B, mg/dL**	0.13 ± 0.14	0.11	0.02	0.86	0.06	0.16

**Table 3:** Blood biochemistry data of the 25 *C. caretta* sub-adult subjects.

Sub-Adults (n.25)						
Parameter	Mean (SD)	Median	Min	Max	10° Percentile	90° Percentile
**Na, mmol/L**	155.85 ± 5.48	158	142.20	162.20	149	161.10
**K, mmol/L**	3.15 ± 0.48	3.10	2.18	3.70	2502	3648
**Cl, mmol/L**	115.06 ± 6.41	116.70	105	131.70	107.46	121.78
**Glu, mg/dL**	123.70 ± 38.09	106.10	55.80	174	77.94	179.40
**ALT, U/L**	5.16 ± 7.48	1	0	26	0	13.60
**AST, U/L**	230.04 ± 192	209	1	923	65.20	436.20
**P, mg/dL**	11.73 ± 14.15	7.95	1.60	66	4174	12,208
**Mg, mg/dL**	9.77 ± 10.26	3.95	1.30	12.39	2.38	27,716
**Ca, mg/dL**	5.41 ± 1.42	5.80	1.78	7.70	3.72	6.62
**ALP, U/L**	22.28 ± 19.80	20	2	84	6	35
**γ-GT, U/L**	1.28 ± 1.06	1.50	0	4	0	2
**TP, g/dL**	3.224 ± 1.73	3	0.30	5.90	1	5
**ALB, g/dL**	1.02 ± 0.60	1105	0	1.70	0.1	1.70
**Urea, mg/dL**	147.63 ± 75.69	133.05	8.60	421.10	86.14	202,832
**CK, U/L**	4017.37 ± 5244	932	1	15,232	152.10	12,194.90
**Fe, µg/dL**	28.88 ± 21	28.50	1	78	5.40	56.40
**LDH, U/L**	230.92 ± 310.77	100.50	0.09	1140	30.80	554.4
**Tot-B, mg/dL**	0.24 ± 0.27	0.15	0.01	1.06	0.07	0.73

**Table 4:** Blood biochemistry data of the 8 *C. caretta* adult subjects.

Adults (n.8)						
Parameter	Mean (SD)	Median	Min	Max	10° Percentile	90° Percentile
**Na, mmol/L**	154.02 ± 4.75	156	147.10	161	148.24	158.90
**K, mmol/L**	3.11 ± 0.85	2.82	1.90	4.36	2.26	4178
**Cl, mmol/L**	107.38 ± 2.76	109.10	104	111.40	104.18	110
**Glu, mg/dL**	126.36 ± 58.90	125.30	47	200	84.26	188.73
**ALT, U/L**	7.50 ± 16.37	2	0	48	1.20	15.80
**AST, U/L**	189.37 ± 76.31	206	163	283	167.20	262
**P, mg/dL**	7.50 ± 3.73	8.80	0.30	11.40	4248	11,127
**Mg, mg/dL**	6.62 ± 7.90	4.50	1.30	25.68	2.56	12,324
**Ca, mg/dL**	6.30 ± 1.65	5.80	4.90	10.20	5.14	7.54
**ALP, U/L**	19.12 ± 12.81	14	4	40	9.40	38.60
**γ-GT, U/L**	2.75 ± 3.61	1	0	11	0	6.10
**TP, g/dL**	3.85 ± 1.88	3.70	0.90	6.40	1.44	5.70
**ALB, g/dL**	1.02 ± 0.95	0.90	0	2.90	0	1.85
**Urea, mg/dL**	130.33 ± 61.71	127.10	75.57	232	93,948	212.33
**CK, U/L**	2697.62 ± 4218.14	682	9	11,121	322.20	8626.20
**Fe, µg/dL**	35 ± 42	15	5	127	7.40	85
**LDH, U/L**	221.12 ± 193.56	170	19	610	22.60	447.60
**Tot-B, mg/dL**	0.19 ± 0.19	0.11	0.07	0.66	0.09	0.37

**Table 5:** Blood biochemistry data of the 8 adult females of *C. caretta* during the reproductive and non-reproductive seasons.

	Reproductive Season (n.4)			Non-Reproductive Season (n.4)		
Parameter	Mean (SD)	Min	Max	Mean (SD)	Min	Max
**Na, mmol/L**	155.52 ± 4.33	152	161	152.52 ± 5.28	147.10	158
**K, mmol/L**	3.36 ± 0.83	2.5	4.36	2.86 ± 0.91	1.90	4.10
**Cl, mmol/L**	109.07 ± 2.05	106.40	111.40	105.70 ± 2.45	104	109.30
**Glu, mg/dL**	100.47 ± 36.29	47	125.30	152.25 ± 70.70	47	200
**ALT, U/L**	13.50 ± 23	2	48	1.50 ± 1	0	2
**AST, U/L**	173.50 ± 108	29	283	205.25 ± 35.11	170	253
**P, mg/dL**	7.61 ± 2.40	3.95	9.10	7.39 ± 5.15	0.30	11.40
**Mg, mg/dL**	3.02 ± 1.93	1.30	5.60	10.22 ± 10.36	4.10	25.68
**Ca, mg/dL**	7 ± 2.16	5.50	10.20	5.60 ± 0.64	4.90	6.40
**ALP, U/L**	27.25 ± 13.60	15	40	11 ± 4.70	4	14
**γ** **-GT, U/L**	5 ± 4	2	11	0.50 ± 0.50	0	1
**TP, g/dL**	4.47 ± 2.44	0.90	6.40	3.22 ± 1.15	1.80	4.50
**ALB, g/dL**	1.17 ± 1.29	0	2.90	0.87 ± 0.61	0	1.40
**Urea, mg/dL**	120.99 ± 64.90	46.77	203.90	139.67 ± 66.65	75.57	232
**CK, U/L**	3230 ± 5270	176	11,121	2165.25 ± 3603	9	7557
**Fe, µg/dL**	58.50 ± 51.28	11	127	11.50 ± 5.50	5	17
**LDH, U/L**	335.25 ± 206.40	170	610	107 ± 100	19	218
**Tot-B, mg/dL**	0.16 ± 0.06	0.10	0.25	0.23 ± 0.28	0.07	0.66

## Data Availability

Data are contained within this article.

## Supplementary Materials

The following supporting information can be downloaded at https://www.mdpi.com/article/10.3390/vetsci11110571/s1: Table S1: Blood biochemistry data of adult *C. caretta* males coming from the Mediterranean Sea.
