# Supplementary material for: Evaluation of Biochemical Parameters in Caretta caretta Sea Turtles
Source: Vet Sci. 2024 Nov 16;11(11):571. doi: 10.3390/vetsci11110571 (PMC11598882; doi:10.3390/vetsci11110571)
Supplement: Supplementary file 1 [file vetsci-11-00571-s001.zip › vetsci-3286858-supplementary.pdf]

veterinary sciences

MDPI

Table S1: Blood biochemistry data of adult *C. caretta* males recovered from the Mediterranean Sea.

Parameter	2022	2023	2024
Na, mmol/L	156	151.4	154.8
K, mmol/L	2.4	3.99	3.97
Cl, mmol/L	113	104.3	118.6
Glu, mg/dL	117	91	188
ALT, U/L	2	2	41
AST, U/L	303	210	393
P, mg/dL	5.1	6.29	8.98
Mg, mg/dL	4	4.31	42.74
Ca, mg/dL	5.18	8.1	7.4
ALP, U/L	17	3	32
γ-GT, U/L	2	1	1
TP, g/dL	1.9	5.6	5.6
ALB, g/dL	1.8	2.4	1.8
UREA, mg/dL	60.1	261.6	174.06
CK, U/L	780	892	8324
Fe, μg/dL	14	60	32
LDH, U/L	239	22	382
Tot- B, mg/dL	0.20	0.13	0.05
